# Prognostic Impact of FoxP3+ Regulatory T Cells in Relation to CD8+ T Lymphocyte Density in Human Colon Carcinomas

**DOI:** 10.1371/journal.pone.0042274

**Published:** 2012-08-06

**Authors:** Harry H. Yoon, Jared M. Orrock, Nathan R. Foster, Daniel J. Sargent, Thomas C. Smyrk, Frank A. Sinicrope

**Affiliations:** 1 Department of Oncology, Mayo Clinic, Rochester, Minnesota, United States of America; 2 Department of Laboratory Medicine and Pathology, Mayo Clinic, Rochester, Minnesota, United States of America; 3 Department of Health Sciences Research, Mayo Clinic, Rochester, Minnesota, United States of America; 4 Department of Medicine, Mayo Clinic, Rochester, Minnesota, United States of America; Baylor University Medical Center, United States of America

## Abstract

**Background:**

T-lymphocyte infiltration into colon carcinomas can influence clinical outcome, and interactions among T cell subsets may be more informative than either subset alone. Our objective was to examine the prognostic impact of tumor-infiltrating FoxP3^+^ regulatory T cells (Tregs) in relation to cytotoxic CD8^+^ T lymphocytes in patients with colon carcinomas characterized by DNA mismatch repair (MMR) status who participated in adjuvant chemotherapy trials.

**Methods:**

FoxP3^+^ and CD8^+^ densities in tumor epithelial and stromal compartments were analyzed by immunohistochemistry and quantified in resected, stage II and III colonic carcinomas (N = 216). Immune marker density was dichotomized at the median and categorized as high *vs* low. MMR status was classified as MMR deficient (dMMR) or proficient (pMMR). Cox models were adjusted for age, stage, and tumor grade.

**Results:**

The density of FoxP3+ infiltration was similar in tumor stroma and epithelia, whereas CD8+ was higher in stroma. The prognostic impact of FoxP3+ and CD8+ T cell infiltration was stronger in stroma *vs* epithelia, and the density of each marker in stroma was independently associated with improved overall survival (OS). However, the impact of FoxP3+ on survival was dependent upon CD8+ density (*P* interaction  = .040). Among CD8+_low_ tumors, FoxP3+_high_ cases had significantly improved OS compared to FoxP3+_low_ cases after adjustment for covariates (hazard ratio 0.43; 95% confidence interval 0.19 to 0.95; P = .030). In contrast, FoxP3+ was not prognostic among CD8+_high_ tumors. FoxP3+ remained prognostic in CD8+_low_ tumors after further adjustment for MMR or *BRAF* ^V600E^ mutation status. Additionally, these immune markers identified a pMMR subgroup with a similarly favorable OS as for dMMR tumors.

**Conclusions:**

The prognostic impact of FoxP3+ and CD8+ T cell density are inter-dependent, whereby FoxP3+ exerts a favorable influence on survival only in colon cancers with low CD8+ infiltration.

## Introduction

Studies in patients with colorectal cancer (CRC) suggest that the density of tumor infiltrating lymphocytes (TILs) strongly influences clinical outcome [1], [2], [3]. High intratumoral density of CD3+ and cytotoxic CD8+ lymphocytes, as well as CD45RO+ memory T cells, has been associated with reduced incidence of tumor metastasis and favorable prognosis [1], [2]. This finding suggests that clinical outcome could be governed, in large part, by the status of the local adaptive immune response. Upon antigenic stimulation, CD8+ T lymphocytes differentiate into effector cells that kill tumor cells by releasing toxic granules such as granzyme B and perforins [4], [5]. We previously found that a high density of TILs in colon cancers is associated with increased tumor cell apoptosis [6]. However, not all studies have found CD8+ infiltration to be prognostic in CRC [7], [8], and recent data suggests that interactions among T cell subsets is a critical factor that controls the host-tumor reaction and predicts disease outcome [9], [10]. An important factor that may block the adaptive immune response are regulatory T cells (CD4+CD25+ Tregs) that express the forkhead box P3 (FoxP3) transcription factor [11]. FoxP3+ Tregs can suppress host-mediated anti-tumor immunity and tumor-specific cytotoxicity, suggesting that Treg depletion is a potential therapeutic strategy [12]. Recent evidence indicates that signaling of the T cell chemoattractant CCL5 can recruit Tregs to tumors and enhance their ability to kill CD8+ T cells, thereby providing a mechanism of immune escape [13]. A high density of infiltrating FoxP3+ Tregs has been shown to be associated with an adverse prognostic impact in some tumor types [10], [11], yet a favorable impact in others, including in colon cancers [8], [14], [15], [16], [17]. Consistent with their role in immune suppression, a high density of intratumoral FoxP3+ Tregs has been associated with poor outcomes in ovarian, pancreatic, and hepatocellular carcinomas [10], [11]. Increased numbers of FoxP3+ Tregs have been detected in colon cancers compared to surrounding unaffected mucosa [18], [19]. However, most, but not all [7], [20], studies in colon cancers have shown a paradoxical and statistically significant association with favorable prognosis [8], [14], [15]. This finding is consistent with the improved prognosis for FoxP3+ T cell infiltration in Hodgkin’s and follicular lymphoma and in head-and-neck carcinomas [16], [17]. Tregs are increased in tissues with ongoing inflammation, such as in inflammatory bowel disease [21], where control of inflammation may inhibit tumor development or progression [22].

**Table 1:** Clinicopathologic Characteristics of Study Population by Immune Marker Density (N = 216).

		FoxP3+ T Cell Densitya,b						CD8+ T Cell Density^a^						
	TOTAL	Stroma			Epithelia			Stroma			Epithelia			
Variable		High	Low	*P*	High	Low	*P*	High	Low	*P*	High	Low	*P*	
**Stage**, N (%)				0.3902			0.1974			0.5734			0.5956	
II	39 (18)	15 (19)	11 (14)		10 (13)	16 (20)		21 (20)	18 (17)		18 (17)	21 (19)		
III	177 (82)	63 (81)	67 (86)		68 (87)	62 (80)		86 (80)	90 (83)		90 (83)	87 (81)		
**Grade**, N (%)^c^				**0.0066**			**0.0174**			0.0574			**0.0046**	
1 or 2	138 (64)	44 (56)	60 (77)		45 (58)	59 (76)		62 (58)	76 (70)		59 (55)	79 (73)		
3 or 4	78 (36)	34 (44)	18 (23)		33 (42)	19 (24)		45 (42)	32 (30)		49 (45)	29 (27)		
**Tumor Site**, N (%)				0.1495			0.4233			0.6301			**0.0064**	
Distal	102 (47)	43 (55)	34 (44)		41 (53)	36 (46)		49 (46)	53 (49)		41 (38)	61 (56)		
Proximal	114 (53)	35 (45)	44 (56)		37 (47)	42 (54)		58 (54)	55 (51)		67 (62)	47 (44)		

a T cell densities were dichotomized at the median.

b FoxP3+ (N = 156).

c Histologic Grade: 1 or 2, well or moderately differentiated; 3 or 4, poor or undifferentiated.

The extent of T lymphocyte infiltration in human CRC has been shown to differ based upon the status of the DNA mismatch repair (MMR) system [23], [24]. CRCs with deficient MMR (dMMR) and microsatellite instability (MSI) are highly immunogenic compared to proficient MMR (pMMR) tumors, the latter of which show chromosomal instability and account for a majority of CRCs. Most dMMR tumors show hypermethylation of the *MLH1* MMR gene promoter and frequent activating mutations in a mutational hotspot within exon 15 of the *BRAF* oncogene (V600E) [23]. An increased density of TILs, including CD3+ and CD8+ lymphocytes, are characteristic of dMMR tumors although studies analyzing the prognostic impact of immune markers have generally not accounted for MMR status [23], [24]. *BRAF* mutation has been reported to be more frequent in stage I to IV human colorectal carcinomas with a high lymphocytic infiltration [25]. In contrast to pMMR cancers, most TILs within dMMR tumors are located within tumor epithelia or in direct contact with tumor cells [26]. Importantly, differences in the tissue location of effector T cell and Treg infiltration within tumor epithelial *vs* stromal compartments may influence their function and prognostic impact [27], [28], yet studies have not adequately addressed this issue, especially for FoxP3+ Tregs [1], [2], [8].

In this study, we determined the density of FoxP3+ and CD8+ T lymphocytes in epithelial and stromal compartments of human colon cancers, and their relationship to MMR status and patient survival rates. We examined resected stage II and III colon cancers from participants in adjuvant chemotherapy trials where treatment was standardized and meticulous long-term clinical follow-up data were collected. We focused on this population, since immune markers may provide prognostic information that could enable risk stratification for adjuvant therapy. Given data indicating that interactions among T cell subsets may be more informative than either marker alone [9], [10], we analyzed the intratumoral densities of FoxP3+ Tregs in relationship to effector CD8+ T cells.

## Methods

### Ethics Statement

The study was approved by the Mayo Clinic institutional review board.

### Patient Specimens

Surgically resected TNM stage II and III primary colon adenocarcinomas (N = 216) were analyzed from three 5-fluorouracil (5-FU)–based adjuvant chemotherapy trials conducted by the Mayo Clinic and the North Central Cancer Treatment Group. Formalin-fixed, paraffin-embedded tumor blocks from a nonrandom subset of study participants with MMR data were used. Tumors at or below the splenic flexure were categorized as distal; the remainder as proximal. Of the 216 patients, 191 were randomized to systemic 5-FU, and the others received surgery alone or 5-FU by portal vein infusion. The number of patients who received each study treatment is as follows: 5-FU and leucovorin (LV) plus levamisole (N = 134; study 91-46-53), 5-FU plus levamisole *vs* 5-FU plus levamisole plus LV (N = 57; study 89-46-51), or portal venous 5-FU *vs* observation (N = 10 *vs* N = 15; study 79-46-04) [29], [30].

**Table 2:** Univariate Associations of Clinicopathologic Variables in Relation to Overall Survival (N = 216).

Clinicopathologic Variable	Hazard Ratio	95% Confidence Interval	*P*
**Age**			
Per year increase	1.02	1.00 to 1.05	**0.0389**
**Sex**			
Men *vs* Women	0.85	0.55 to 1.31	0.4612
**Stage**			
III *vs* II	2.03	1.01 to 4.07	**0.0429**
**Histologic Grade**			
Poor or undifferentiated *vs*	1.58	1.02 to 2.46	**0.0382**
Well or moderate			
**Tumor Site**			
Proximal vs Distal	0.82	0.52 to 1.27	0.3728
**CD8+ T-cell Density** a			
** Epithelial**			
High *vs* Low	0.68	0.43 to 1.08	0.0978
**Stromal**			
High *vs* Low	0.56	0.36 to 0.87	**0.0091**
**Combined Epithelial-Stromal**			
High-High *vs* Low-Low	0.44	0.24 to 0.80	**0.0075** c
**FoxP3+ T-cell Density** a,b			
**Epithelial**			
High *vs* Low	0.75	0.43 to 1.31	0.3102
**Stromal**			
High *vs* Low	0.69	0.40 to 1.18	0.1751
**Combined Epithelial-Stromal**			
High-High *vs* Low-Low	0.58	0.29 to 1.18	0.1354 c

a CD8+ and FoxP3+ densities were dichotomized at the median.

b FoxP3+ (N = 156).

c Wald chi-square *P* value.

### Immunohistochemical Detection of Immune Markers

Immunohistochemistry (IHC) was performed using tissue microarrays (TMA) containing three 0.6-mm diameter tumor cores per specimen, as previously described [31]. TMAs included normal liver, tonsil, and placenta as controls and navigation markers (Fig. S1). Primary antibodies included anti-FoxP3 mouse monoclonal (ab20034, Abcam, Cambridge, MA; 1∶50; 30 min.) and anti-CD8 mouse monoclonal antibodies (clone C8/144B; Dako; 1∶20; 30 min). Human tonsil was used as a positive control; for a negative control, the primary antibody was omitted but all other steps were followed.

**Figure 1:**
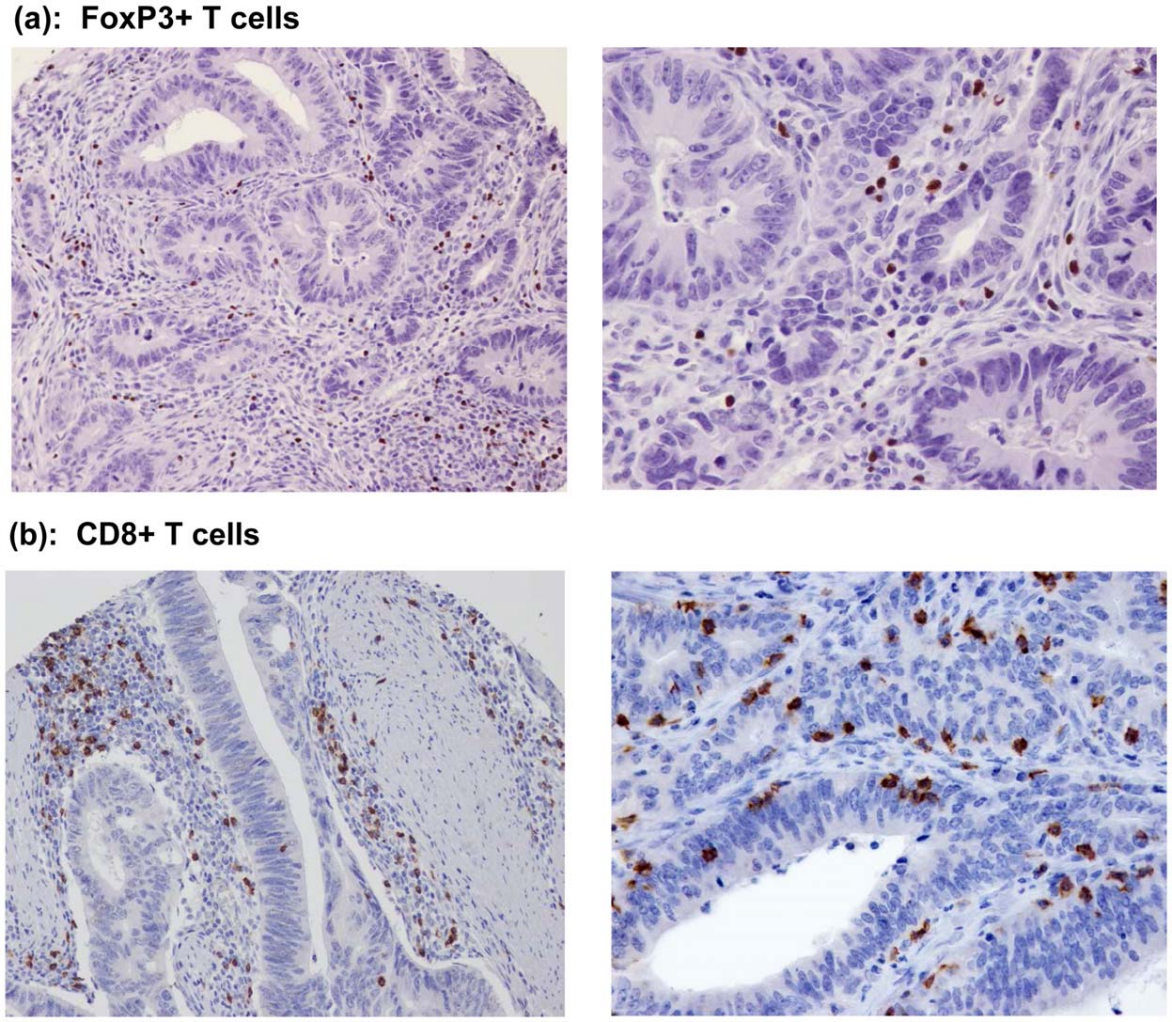
Immune marker expression in colon carcinomas. Expression of FoxP3+ (a) and cytotoxic CD8+ (b) proteins in T lymphocytes, determined by immunohistochemistry, is shown infiltrating the tumor stroma and epithelia of resected colon carcinomas (*left*, 20× objective; *right*, 40× objective).

### Analysis and Quantification of Immune Markers

TMA slides were imaged using the Slide Scanner system (Bacus Laboratories Inc., Lombard, IL), as described [32]. For CD8+ (N = 216) and FoxP3+ (N = 156), positively-stained cells were counted within each core (20× magnification) in epithelial (within the tumor cell nests or directly contacting a tumor cell) and stromal compartments [20], [33]. Density measurements were recorded as the number of positive cells per tumor core (cells per 0.28 mm^2^ surface area) [20], [34]. Scoring criteria were established by two pathologists (J.M.O; T.C.S.) using randomly selected cases, then all specimens were scored without knowledge of clinical information.

**Table 3:** Multivariable Cox Models for Overall Survival Examining CD8+ and FoxP3+ T-cell Density^a^.

	Not Adjusted for MMR			Adjusted for MMR		
Immune Markers^b^	HR	95% CI	p	HR	95% CI	p
***CD8+ cytotoxic T cells***						
** Model A**						
Epithelial (High *vs* Low)	0.61	0.38 to 0.96	**0.0323**	0.63	0.38 to 1.03	0.063
** Model B**						
Stromal (High *vs* Low)	0.52	0.33 to 0.83	**0.0049**	0.56	0.34 to 0.93	**0.021**
***FoxP3+ T cells***						
** Model A**						
Epithelial (High *vs* Low)	0.58	0.32 to 1.05	0.073	0.51	0.26 to 1.00	**0.045**
** Model B**						
Stromal (High *vs* Low)	0.61	0.35 to 1.06	0.077	0.44	0.23 to 0.83	**0.010**

a Each model is adjusted for age, stage, grade.

b CD8+ and FoxP3+ densities were dichotomized at the median.

Abbreviations: MMR, mismatch repair.

### DNA MMR and BRAF Mutation Status

The status of the DNA MMR system was determined by polymerase chain reaction amplification of microsatellite loci in microdissected tumor-enriched paraffin tissue using 5–11 microsatellite markers, as previously described [20], [30], [35], [36]. Data were available on 183 (85%) patients. MSI results were interpreted as follows: (1) microsatellite stable (MSS) with no MSI at any of the loci examined, (2) low instability (MSI-L; <30% of the loci demonstrating MSI), or (3) high instability (MSI-H; ≥30% of the loci demonstrating MSI) [35], [36]. Tumors were considered to show dMMR if MSI-H (studies 89-46-51 and 79-46-04) or had instability at the BAT26 mononucleotide locus coupled with absent expression of an MMR protein (MLH1, MSH2, MSH6) (study 91-46-53) [20], [30]. Proficient MMR included tumors showing MSS/MSI-L or lacking instability at BAT26 with intact MMR proteins. Testing for *BRAF* ^V600E^ mutation status in exon 15 had been determined as previously described using genomic DNA extracted from formalin-fixed, paraffin-embedded tumor tissue (NCCTG 91-46-53; N = 125) [37].

**Figure 2:**
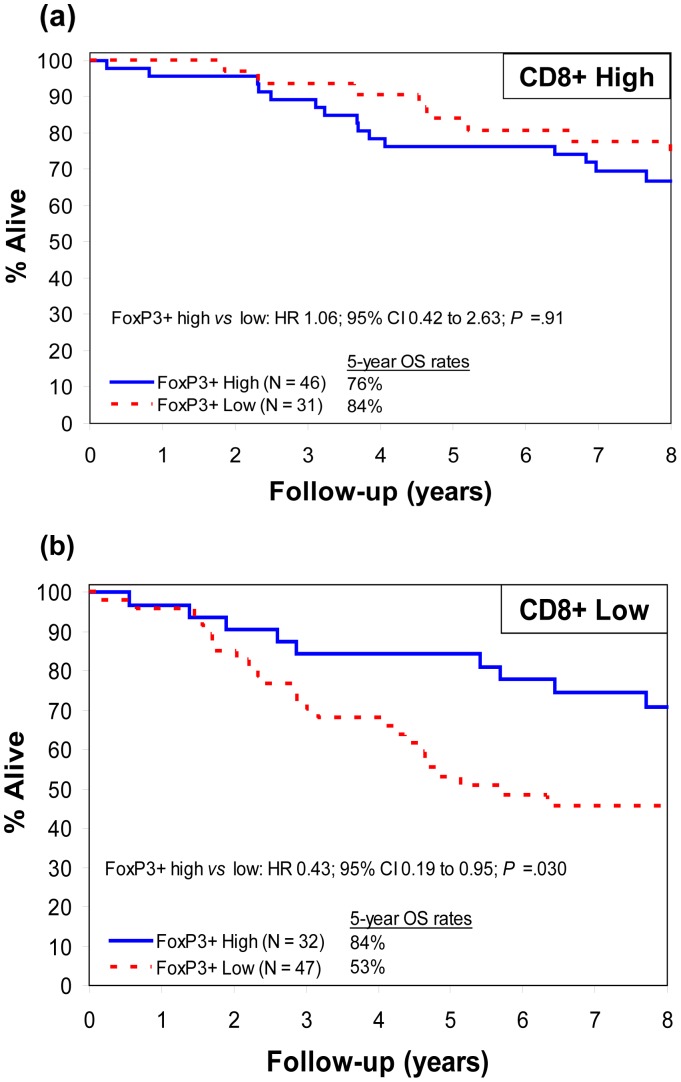
Patient survival according to CD8+ and FoxP3+ T-cell density. Overall survival (OS) in resected colon carcinomas with (a) high *vs* (b) low density of cytotoxic CD8+ T-cell infiltration in tumor stroma according to FoxP3+ T-cell density in tumor stroma (*P* for interaction  = .040). Hazard ratios (HR) are adjusted for age, stage, and tumor grade.

### Statistical Analysis

Chi-square tests were used for categorical variables, and the Wilcoxon rank-sum test was used to associate continuous marker densities with 2-level categorical data. The Wilcoxon signed-rank test was used to compare stroma and epithelia densities for each immune marker. T-cell densities were divided at the median for comparisons with clinical variables, including overall survival (OS). OS, censored at 8 years, was calculated from randomization to the date of death or last contact. Within the study population, 81 patients died, and 135 are still alive (global survival rate = 62.5%). Median follow-up in the 135 alive patients was 8 years (range: 4.7–8.0), since the data was censored at 8 years. Kaplan–Meier methodology and Cox proportional hazards models were used, stratified by parent study, for survival analysis. Score and likelihood ratio test P values were used in univariate and multivariate models, respectively. The likelihood ratio test was used to test for interactions between variables. Multivariable analyses included adjustment for age, stage, grade and, when noted, MMR. Statistical tests were 2-sided with P<.05 considered statistically significant. Analyses were performed using SAS software (Cary, NC).

## Results

### Study Population

TNM stage II and III primary colon adenocarcinomas (N = 216) were analyzed from participants in 5-FU-based adjuvant trials. Median age was 64 years (range 26–83), and 114 (53%) patients were male. Other characteristics are shown in Table 1. Compared to pMMR (161 [88%]) tumors, cases with dMMR (22 [12%]) were significantly more likely to be proximal (P = .0007), high grade (P = .0005) (Table S1), and to carry *BRAF* ^V600E^ mutations (50% vs. 12%; P = .0004).

In the overall study cohort, we found that 19 cases carried the *BRAF* ^V600E^ mutation (15.2%). Study and parent cohorts had similar dMMR rates.

Age, stage, and grade were significantly associated with OS (Table 2). Five-year OS rates were similar among study and parent cohorts (70% *vs* 68%; P = .467). Among dMMR tumors, 5-year OS rates were also similar in the study and parent cohorts. Within the study cohort, the favorable impact of dMMR (*vs* pMMR) on OS was of borderline statistical significance after adjustment for covariates (HR 0.49; 95% CI 0.22 to 1.11; P = .064), but was highly significant in the parent cohort (HR 0.61; 95% CI 0.42 to 0.89; P = .0066).

**Table 4:** Multivariable Cox Models for Overall Survival Examining Stromal FoxP3+ T-cell Density Stratified by CD8+ T-cell Density^a^.

	Total Cohort			Mismatch Repair Proficient Subgroup (pMMR)^b^		
	HR	95% CI	p	HR	95% CI	p
***CD8+ High Tumors (N = 77)*** c						
FoxP3+ (High *vs* Low)	1.06	0.42 to 2.63	0.907	0.43	0.14 to 1.31	0.138
Age (per year increase)	1.05	1.00 to 1.10	**0.040**	1.03	0.97 to 1.08	0.312
Stage (III *vs* II)	1.52	0.44 to 5.22	0.483	3.44	0.43 to 27.73	0.174
Grade (1, 2 *vs* 3, 4)	0.37	0.15 to 0.90	**0.026**	0.27	0.09 to 0.87	**0.026**
***CD8+ Low Tumors (N = 79)*** c						
FoxP3+ (High *vs* Low)	0.43	0.19 to 0.95	**0.030**	0.30	0.11 to 0.81	**0.012**
Age (per year increase)	0.99	0.96 to 1.03	0.739	0.96	0.92 to 1.01	0.124
Stage (III *vs* II)	0.81	0.32 to 2.08	0.668	0.84	0.29 to 2.38	0.741
Grade (1, 2 *vs* 3, 4)	0.40	0.18 to 0.89	**0.030**	0.29	0.10 to 0.82	**0.026**

a CD8+ and FoxP3+ densities were dichotomized at the median.

b pMMR (N = 119).

c Within each model, hazard ratios and p values are adjusted for all variables shown.

### Immune Cell Density

We analyzed the expression and location of infiltrating cytotoxic CD8+ T cells and FoxP3+ regulatory T cells within colon carcinomas (Figure 1). Staining was analyzed and compared within epithelial and stromal compartments. CD8+ density was significantly higher in stroma *vs* epithelia, whereas FoxP3+ was similar (Table S2).

### Immune Marker Density in Relation to Clinicopathological Variables

For analysis of immune markers in relation to clinical variables, we dichotomized immune marker density at the median. We found that high *vs* low CD8+ densities were associated with high-grade histology (Table 1). Similar results were found for FoxP3+. Within tumor epithelia, high CD8+ density was significantly associated with proximal tumor site. T cell densities were not significantly associated with stage, number of malignant nodes, or T stage. CD8+ and FoxP3+ were positively associated with one another.

Analysis of immune markers in relation to MMR status revealed that CD8+ density was significantly higher in epithelia of dMMR *vs* pMMR cases (median 41 *vs* 10 cells; P<.0001), yet did not differ in tumor stroma. For FoxP3+, location did not differ by MMR status. No statistically significant associations were found between *BRAF* status and CD8+ density (P = .26), or between *BRAF* and FoxP3+ density (P = .83).

**Figure 3:**
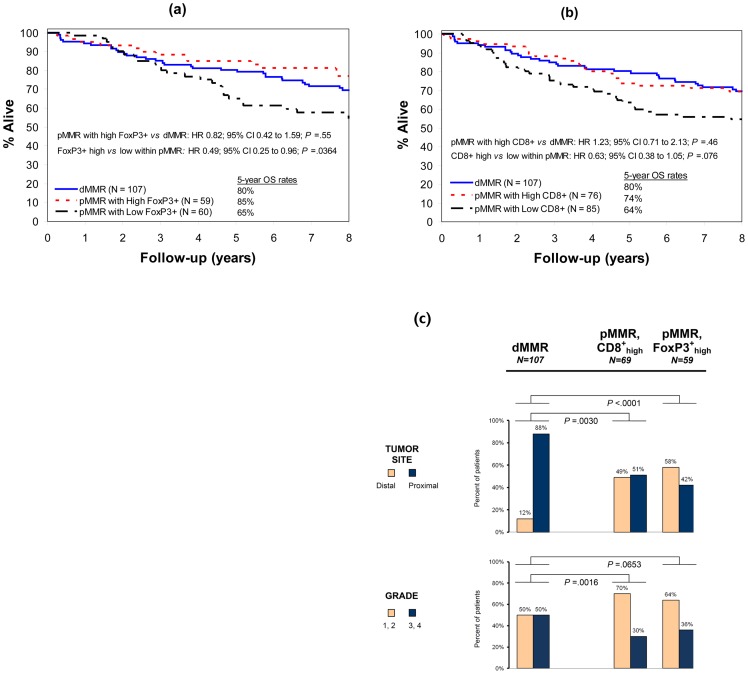
Patient survival and comparison of clinicopathologic variables by MMR status and T-cell density. Overall survival (OS) in deficient MMR (dMMR) *vs* proficient MMR (pMMR) colon carcinomas according to the density of FoxP3+ (a) or CD8+ (b) T lymphocytes in tumor stroma, showing similar OS among cases with dMMR *vs* pMMR with high densities of CD8+ or FoxP3+. Comparison of clinicopathological variables (c) between dMMR *vs* CD8+_high_ or FoxP3+_high_ pMMR cancers demonstrates differences in histologic grade and tumor site. Grade: 1 or 2, well- or moderately differentiated; 3 or 4, poor or undifferentiated.

### T-cell Infiltration and Prognosis

Univariately, high CD8+ T cell density was significantly associated with improved OS in tumor stroma (P = .0091), but not in epithelia (P = .0978; Table 2). Neither FoxP3+ in stroma nor epithelia was prognostic. Analysis of CD8+ density in stroma plus epithelia suggested a stronger prognostic impact compared to analyzing either tumor compartment alone (P = .0075; Table 2).

In multivariable models that included age, stage, and grade, high CD8+ density in stroma (P = .0049) and in epithelia (P = .0323) were significantly associated with OS (Table 3). For FoxP3+, high epithelial or stromal density had borderline associations with favorable OS. After adjustment for MMR status, the favorable prognostic impact of CD8+ density was weakened, whereas the impact of FoxP3+ density was strengthened. For both FoxP3+ and CD8+, MMR-adjusted models showed greater prognostic impact for stromal (*vs* epithelial) infiltration (Table 3). Adjustment for *BRAF* status did not significantly alter the findings shown in the multivariable models.

In exploratory analysis, evaluation of the immune markers in stroma plus epithelia showed greater impact on OS compared to either compartment alone. In this regard, high FoxP3+ density in both compartments was associated with substantially improved OS compared to low density in both (HR 0.29 adjusted for covariates and MMR; 95% CI 0.12 to 0.71; P = .0064), and results were similar for CD8+ (HR 0.38; 95% CI 0.19 to 0.74; P = .0048).

### Interdependence of FoxP3+ and CD8+ T Cells on Prognosis

To determine whether the prognostic impact of FoxP3+ and CD8+ was inter-dependent, we tested for their interaction and found a significant relationship for OS (P for interaction  = .040; P*_adjusted_*  = .07). FoxP3+ density stratified by CD8+ revealed that, among CD8+_high_ tumors, OS was similarly favorable among FoxP3+_high_ *vs* FoxP3+_low_ tumors (adjusted P = .91; Figure 2a; Table 4). Among CD8+_low_ tumors, however, OS was significantly improved in FoxP3+_high_ *vs* FoxP3+_low_ cases (HR 0.43; 95% CI 0.19 to 0.95; P = .030; Figure 2b; Table 4). Notably, the 5-year survival rate in the CD8+_low_FoxP3+_high_ group (84% [95% CI 73–98]) was similar to that of CD8+_high_ tumors, and was lowest in cases with low densities of both markers (53% [95% CI 41–70]) (Figure 2a and 2b). Importantly, the associations of FoxP3+ with OS, when stratified by CD8+, were similar after further adjustment for MMR status.

We also examined the converse situation where CD8+ T cell density was stratified by FoxP3+ density. We found that high CD8+ density was not prognostic among FoxP3+_high_ tumors (adjusted P = .530), but was associated with improved OS among FoxP3+_low_ tumors (HR 0.34; 95% CI 0.15 to 0.77; P = .0060). The association of CD8+ with OS became non-significant after further adjustment for MMR or BRAF.

### Immune Markers in Proficient vs Deficient MMR Tumors

To remove potential confounding by dMMR, we determined the prognostic impact of the FoxP3+ and CD8+ in pMMR cases and found consistent results (Table 4). We sought to identify a favorable prognostic group using these immune markers within pMMR tumors. FoxP3+_high_ or CD8+_high_ cases that were pMMR showed similarly favorable OS as did 107 dMMR cases from the parent cohorts (Figure 3a–3b). Interestingly, pMMR cases with FoxP3+_high_ or CD8+_high_ showed differences in tumor site and histologic grade compared to phenotypic characteristics of dMMR cases [38] (Figure 3c).

## Discussion

Given data suggesting that interactions among T lymphocyte subsets may regulate the host-mediated anti-tumor reaction [9], [10], we examined the association of FoxP3+ and CD8+ T cells with patient prognosis in stage II and III colon cancers from participants in adjuvant chemotherapy studies. We found that FoxP3+ and CD8+ densities in tumor stroma were independently associated with patient survival in multivariable models. However, their prognostic impact differed significantly based on the relative density of the other, suggesting that these markers are inter-dependent and should not be directly compared within the same model. We made the novel observation that the FoxP3+ T-cell density independently predicts a favorable OS only when the density of CD8+ infiltration is low. When CD8+ density was high, patient survival was favorable regardless of the level of FoxP3+ infiltration. The converse was also true in that the favorable prognostic impact of CD8+ infiltration was most evident when FoxP3+ density was low. Of note, the best survival rates were observed in cases with high density of either marker, and lowest in cases with low density of both markers. These data suggest that CD8+ and FoxP3+ T cells can interact to regulate the anti-tumor immune response, whereby redundancy in their prognostic impact may exist when there is high-level infiltration of both. However, a microenvironment with low cytotoxic T cells enables the ability of high FoxP3+ density to exert a favorable prognostic influence.

We demonstrate a paradoxically favorable effect of FoxP3+ T cells on patient survival that is supported by a study in the *Apc^MIN/+^* mouse model of intestinal polyposis where adoptive transfer of Tregs was able to regress established tumors [39]. CRCs are heavily infiltrated by innate inflammatory immune cells (*e.g.,* tumor-associated macrophages, neutrophils) [22], and studies in mice have shown that adoptive transfer of Tregs can inhibit bacteria-driven inflammation and subsequent colon cancer [40], [41]. These data raise the possibility that FoxP3+ T lymphocytes may preferentially suppress T-cell immune responses driven by microbial rather than tumor-associated antigens [11], and could account for their observed favorable prognostic impact in tumors rich in microbial pathogens, such as cancers of the colon and oral cavity [17], [42], [43]. Evidence also suggests that Tregs may have differential effects depending upon the extent of tumor development/progression. In preclinical studies, depletion of FoxP3+Tregs in mice early in tumor progression enabled immune rejection whereas no therapeutic effect was seen with established tumors [44]. A recent study in human CRC and in a murine polyposis model found that the interaction of Tregs with mast cells can reverse the anti-inflammatory function of Tregs, achieving a pathologic shift in the natural role of Tregs from controlling acute inflammation to promoting inflammation [45]. This Treg skewing is consistent with the known plasticity of Tregs and was associated with their reduced expression of IL10, a cytokine that is critical for maintaining Treg suppressor function [45]. In addition, tumor-infiltrating Tregs were found to share characteristics of T_H_17 proinflammatory T cells [45], and a T_H_17-biased microenvironment has been commonly linked with protective effector T-cell responses in cancer [46], [47]. Potential mechanisms by which Tregs can eradicate tumor cells are suggested by the observation that Tregs can express cytotoxic molecules such as granzyme and perforin, and have been shown to induce monocyte death [48]. Furthermore, adoptive immunotherapy with CD4^+^CD25^+^ Tregs has been shown to decrease tumor multiplicity through induction of apoptosis in intestinal tumors, which supports the possibility that in certain contexts, Tregs can directly or indirectly eradicate tumor cells [39]. Human FoxP3+ Tregs have also been shown to possess an effector differentiation program resulting in the production of IL-17 [49] that was shown to inhibit tumor growth by a T cell-dependent mechanism [50].

Immune marker density in tumor stroma yielded stronger prognostic information than in epithelia. Furthermore, analyzing both compartments together was found to strengthen the association of each immune marker with prognosis compared to analysis of either alone (data not shown). We also determined whether the prognostic impact of CD8+ or FoxP3+ in epithelia and stroma were impacted by inclusion of dMMR tumors. Of note, CD8+ density in tumor epithelium, but not stroma, was higher in dMMR compared to pMMR tumors, whereas no difference was seen for FoxP3+. In survival analyses, adjustment for MMR status was shown to weaken the favorable influence of CD8+_high_ in both epithelial and stromal compartments, but was found to strengthen the prognostic impact of FoxP3+. While *BRAF* mutation has been associated with an increased immune cell infiltration in colorectal carcinomas [25], adjustment for *BRAF* mutational status in our study did not significantly impact the findings shown in multivariable models. Given the potential confounding influence of dMMR tumors with their pronounced T cell infiltration and favorable prognosis [23], [24], we excluded them to examine the immune markers in a pMMR subset. Within pMMR cases, FoxP3+ remained favorably prognostic in CD8+_low_, but not CD8+_high_, tumors. However, CD8+ was not prognostic when stratified by FoxP3+ (data not shown). These findings suggest that the prognostic impact of FoxP3+ is independent of dMMR, in contrast to CD8+ T cells. Since the larger population of pMMR cases is considered poorly immunogenic which may contribute to worse clinical outcome vs dMMR [18], [51], we sought to determine if high densities of our immune markers could identify a favorable prognostic group within pMMR tumors. Importantly, we found that pMMR tumors with FoxP3+_high,_ and to a lesser extent CD8+_high,_ had similarly favorable survival as did dMMR cases despite dissimilar clinicopathologic features. If confirmed in a larger cohort, these data suggest that immune markers could identify a pMMR subgroup with a favorable prognosis in whom adjuvant chemotherapy may be unnecessary, similar to the situation for stage II dMMR tumors [52].

While FoxP3 is the most specific marker for identifying Tregs [53], important caveats exist. Activated CD4^+^CD25^−^ effector T cells can transiently express FoxP3+ that may or may not be associated with the acquisition of suppressor functions [54]. Furthermore, there are CD8+CD25+FoxP3+ T cells that appear uncommon in CRCs, and can suppress the anti-tumor immune response [11]. Data indicate that *FoxP3* demethylation identifies Tregs with stable FoxP3 expression, and is more specific than mRNA or protein expression in discriminating Tregs from activated FoxP3+ conventional T cells [55]. Further study of *FOXP3* demethylation and functional studies of FoxP3+ cells in CRCs are eagerly awaited.

Our finding that immune markers were independent prognostic variables in a modest-sized patient cohort underscores their robust prognostic impact and potential clinical utility. While our study population represents a subset of patients from the parent studies, our study cohort had similar OS rates as did the parent cohorts where dMMR was significantly associated with favorable outcome. A strength of our study population is the meticulous collection of long-term survival data from adjuvant trials. Since most of the patients in our study received adjuvant 5-FU-based chemotherapy, we cannot exclude the possibility that the prognostic impact of FoxP3+ and CD8+ T cell density may differ among patients who did not receive chemotherapy. In this regard, the predictive impact of our T-cell markers is unknown and awaits further study. While we examined three tumor cores per patient from tissue regions that were considered representative, we acknowledge that TMAs have limitations for analysis of tumor heterogeneity. Because our stratified analysis was not planned *a priori*, it will be important to confirm our findings in an independent cohort.

In conclusion, we found that the prognostic impact of FoxP3+ and CD8+ T cell density are inter-dependent, whereby FoxP3+ exerted a favorable influence on survival only in colon cancers with low CD8+ infiltration. Therefore, these data suggest a novel inter-relationship between these immune markers in colon cancers whereby the prognostic impact of FoxP3+ T cells is enhanced in a background of low cytotoxic CD8+ cells.

## Supporting Information

Figure S1 **Control images for CD8+ and FoxP3+ staining.** Normal human liver is shown as a negative control for CD8+ (a) and FoxP3+ (b) T cell immunostaining, and tonsil tissue (c) was stained as a positive control for FoxP3+ T cells (20× magnification).
(PDF)

Table S1 **Comparison of Clinicopathologic Variables by Mismatch Repair Status.**
(DOC)

Table S2 **Density of T Lymphocyte Infiltration in Different Locations per Tumor Core (0.28 mm^2^ surface area) in Colon Cancer (N  = 216).**
(DOC)

## References

[1] Galon J, Costes A, Sanchez-Cabo F, Kirilovsky A, Mlecnik B, et al (2006) Type, density, and location of immune cells within human colorectal tumors predict clinical outcome. Science 313: 1960–1964. 17008531 10.1126/science.1129139

[2] Pages F, Berger A, Camus M, Sanchez-Cabo F, Costes A, et al (2005) Effector memory T cells, early metastasis, and survival in colorectal cancer. N Engl J Med 353: 2654–2666. 16371631 10.1056/NEJMoa051424

[3] Guidoboni M, Gafa R, Viel A, Doglioni C, Russo A, et al (2001) Microsatellite instability and high content of activated cytotoxic lymphocytes identify colon cancer patients with a favorable prognosis. Am J Pathol 159: 297–304. 11438476 10.1016/S0002-9440(10)61695-1 PMC1850401

[4] Morishima N, Owaki T, Asakawa M, Kamiya S, Mizuguchi J, et al (2005) Augmentation of effector CD8+ T cell generation with enhanced granzyme B expression by IL-27. J Immunol 175: 1686–1693. 16034109 10.4049/jimmunol.175.3.1686

[5] Pearce EL, Mullen AC, Martins GA, Krawczyk CM, Hutchins AS, et al (2003) Control of effector CD8+ T cell function by the transcription factor Eomesodermin. Science 302: 1041–1043. 14605368 10.1126/science.1090148

[6] Sinicrope FA, Rego RL, Garrity-Park MM, Foster NR, Sargent DJ, et al (2007) Alterations in cell proliferation and apoptosis in colon cancers with microsatellite instability. Int J Cancer 120: 1232–1238. 17187355 10.1002/ijc.22429

[7] Nosho K, Baba Y, Tanaka N, Shima K, Hayashi M, et al (2010) Tumour-infiltrating T-cell subsets, molecular changes in colorectal cancer, and prognosis: cohort study and literature review. J Pathol 222: 350–366. 20927778 10.1002/path.2774 PMC3033700

[8] Salama P, Phillips M, Grieu F, Morris M, Zeps N, et al (2009) Tumor-infiltrating FOXP3+ T regulatory cells show strong prognostic significance in colorectal cancer. J Clin Oncol 27: 186–192. 19064967 10.1200/JCO.2008.18.7229

[9] Sato E, Olson SH, Ahn J, Bundy B, Nishikawa H, et al (2005) Intraepithelial CD8+ tumor-infiltrating lymphocytes and a high CD8+/regulatory T cell ratio are associated with favorable prognosis in ovarian cancer. Proc Natl Acad Sci U S A 102: 18538–18543. 16344461 10.1073/pnas.0509182102 PMC1311741

[10] Gao Q, Qiu SJ, Fan J, Zhou J, Wang XY, et al (2007) Intratumoral balance of regulatory and cytotoxic T cells is associated with prognosis of hepatocellular carcinoma after resection. J Clin Oncol 25: 2586–2593. 17577038 10.1200/JCO.2006.09.4565

[11] Ladoire S, Martin F, Ghiringhelli F (2011) Prognostic role of FOXP3+ regulatory T cells infiltrating human carcinomas: the paradox of colorectal cancer. Cancer Immunol Immunother 60: 909–918. 21644034 10.1007/s00262-011-1046-y PMC11028605

[12] Phan V, Disis ML (2008) Tumor stromal barriers to the success of adoptive T cell therapy. Cancer Immunol Immunother 57: 281–283. 17646987 10.1007/s00262-007-0356-6 PMC11030862

[13] Chang LY, Lin YC, Mahalingam J, Huang CT, Chen TW, et al (2012) Tumor-derived chemokine CCL5 enhances TGF-beta-mediated killing of CD8(+) T cells in colon cancer by T-regulatory cells. Cancer research 72: 1092–1102. 22282655 10.1158/0008-5472.CAN-11-2493

[14] Frey DM, Droeser RA, Viehl CT, Zlobec I, Lugli A, et al (2010) High frequency of tumor-infiltrating FOXP3(+) regulatory T cells predicts improved survival in mismatch repair-proficient colorectal cancer patients. Int J Cancer 126: 2635–2643. 19856313 10.1002/ijc.24989

[15] Correale P, Rotundo MS, Del Vecchio MT, Remondo C, Migali C, et al (2010) Regulatory (FoxP3+) T-cell tumor infiltration is a favorable prognostic factor in advanced colon cancer patients undergoing chemo or chemoimmunotherapy. Journal of immunotherapy 33: 435–441. 20386463 10.1097/CJI.0b013e3181d32f01 PMC7322625

[16] Lee AM, Clear AJ, Calaminici M, Davies AJ, Jordan S, et al (2006) Number of CD4+ cells and location of forkhead box protein P3-positive cells in diagnostic follicular lymphoma tissue microarrays correlates with outcome. J Clin Oncol 24: 5052–5059. 17033038 10.1200/JCO.2006.06.4642

[17] Badoual C, Hans S, Rodriguez J, Peyrard S, Klein C, et al (2006) Prognostic value of tumor-infiltrating CD4+ T-cell subpopulations in head and neck cancers. Clin Cancer Res 12: 465–472. 16428488 10.1158/1078-0432.CCR-05-1886

[18] Le Gouvello S, Bastuji-Garin S, Aloulou N, Mansour H, Chaumette MT, et al (2008) High prevalence of Foxp3 and IL17 in MMR-proficient colorectal carcinomas. Gut 57: 772–779. 17965063 10.1136/gut.2007.123794

[19] Ling KL, Pratap SE, Bates GJ, Singh B, Mortensen NJ, et al (2007) Increased frequency of regulatory T cells in peripheral blood and tumour infiltrating lymphocytes in colorectal cancer patients. Cancer Immun 7: 7. 17388261 PMC2935744

[20] Sinicrope FA, Rego RL, Ansell SM, Knutson KL, Foster NR, et al (2009) Intraepithelial effector (CD3+)/regulatory (FoxP3+) T-cell ratio predicts a clinical outcome of human colon carcinoma. Gastroenterology 137: 1270–1279. 19577568 10.1053/j.gastro.2009.06.053 PMC2873775

[21] Maul J, Loddenkemper C, Mundt P, Berg E, Giese T, et al (2005) Peripheral and intestinal regulatory CD4+ CD25(high) T cells in inflammatory bowel disease. Gastroenterology 128: 1868–1878. 15940622 10.1053/j.gastro.2005.03.043

[22] Terzic J, Grivennikov S, Karin E, Karin M (2010) Inflammation and colon cancer. Gastroenterology 138: 2101–2114 e2105. 10.1053/j.gastro.2010.01.058 20420949

[23] Sinicrope FA (2010) DNA mismatch repair and adjuvant chemotherapy in sporadic colon cancer. Nat Rev Clin Oncol 7: 174–177. 20190798 10.1038/nrclinonc.2009.235 PMC3767984

[24] Jass JR (2007) Classification of colorectal cancer based on correlation of clinical, morphological and molecular features. Histopathology 50: 113–130. 17204026 10.1111/j.1365-2559.2006.02549.x

[25] Ogino S, Nosho K, Irahara N, Meyerhardt JA, Baba Y, et al (2009) Lymphocytic reaction to colorectal cancer is associated with longer survival, independent of lymph node count, microsatellite instability, and CpG island methylator phenotype. Clin Cancer Res 15: 6412–6420. 19825961 10.1158/1078-0432.CCR-09-1438 PMC2771425

[26] Takemoto N, Konishi F, Yamashita K, Kojima M, Furukawa T, et al (2004) The correlation of microsatellite instability and tumor-infiltrating lymphocytes in hereditary non-polyposis colorectal cancer (HNPCC) and sporadic colorectal cancers: the significance of different types of lymphocyte infiltration. Jpn J Clin Oncol 34: 90–98. 15067103 10.1093/jjco/hyh018

[27] Quinn E, Hawkins N, Yip YL, Suter C, Ward R (2003) CD103+ intraepithelial lymphocytes–a unique population in microsatellite unstable sporadic colorectal cancer. Eur J Cancer 39: 469–475. 12751377 10.1016/s0959-8049(02)00633-0

[28] Di Sabatino A, Ciccocioppo R, D’Alo S, Parroni R, Millimaggi D, et al (2001) Intraepithelial and lamina propria lymphocytes show distinct patterns of apoptosis whereas both populations are active in Fas based cytotoxicity in coeliac disease. Gut 49: 380–386. 11511560 10.1136/gut.49.3.380 PMC1728419

[29] Allegra CJ, Parr AL, Wold LE, Mahoney MR, Sargent DJ, et al (2002) Investigation of the prognostic and predictive value of thymidylate synthase, p53, and Ki-67 in patients with locally advanced colon cancer. J Clin Oncol 20: 1735–1743. 11919229 10.1200/JCO.2002.07.080

[30] O’Connell MJ, Sargent DJ, Windschitl HE, Shepherd L, Mahoney MR, et al (2006) Randomized clinical trial of high-dose levamisole combined with 5-fluorouracil and leucovorin as surgical adjuvant therapy for high-risk colon cancer. Clin Colorectal Cancer 6: 133–139. 16945169 10.3816/ccc.2006.n.030

[31] Sinicrope FA, Rego RL, Okumura K, Foster NR, O’Connell MJ, et al (2008) Prognostic impact of bim, puma, and noxa expression in human colon carcinomas. Clin Cancer Res 14: 5810–5818. 18794091 10.1158/1078-0432.CCR-07-5202 PMC2948480

[32] Goode EL, Chenevix-Trench G, Hartmann LC, Fridley BL, Kalli KR, et al (2011) Assessment of hepatocyte growth factor in ovarian cancer mortality. Cancer Epidemiol Biomarkers Prev 20: 1638–1648. 21724856 10.1158/1055-9965.EPI-11-0455 PMC3153603

[33] Mahmoud SM, Paish EC, Powe DG, Macmillan RD, Grainge MJ, et al (2011) Tumor-infiltrating CD8+ lymphocytes predict clinical outcome in breast cancer. J Clin Oncol 29: 1949–1955. 21483002 10.1200/JCO.2010.30.5037

[34] Mlecnik B, Tosolini M, Kirilovsky A, Berger A, Bindea G, et al (2011) Histopathologic-based prognostic factors of colorectal cancers are associated with the state of the local immune reaction. J Clin Oncol 29: 610–618. 21245428 10.1200/JCO.2010.30.5425

[35] Halling KC, French AJ, McDonnell SK, Burgart LJ, Schaid DJ, et al (1999) Microsatellite instability and 8p allelic imbalance in stage B2 and C colorectal cancers. J Natl Cancer Inst 91: 1295–1303. 10433618 10.1093/jnci/91.15.1295

[36] Boland CR, Thibodeau SN, Hamilton SR, Sidransky D, Eshleman JR, et al (1998) A National Cancer Institute Workshop on Microsatellite Instability for cancer detection and familial predisposition: development of international criteria for the determination of microsatellite instability in colorectal cancer. Cancer research 58: 5248–5257. 9823339

[37] French AJ, Sargent DJ, Burgart LJ, Foster NR, Kabat BF, et al (2008) Prognostic significance of defective mismatch repair and BRAF V600E in patients with colon cancer. Clinical cancer research : an official journal of the American Association for Cancer Research 14: 3408–3415. 18519771 10.1158/1078-0432.CCR-07-1489 PMC2674786

[38] Hewish M, Lord CJ, Martin SA, Cunningham D, Ashworth A (2010) Mismatch repair deficient colorectal cancer in the era of personalized treatment. Nat Rev Clin Oncol 7: 197–208. 20177404 10.1038/nrclinonc.2010.18

[39] Erdman SE, Sohn JJ, Rao VP, Nambiar PR, Ge Z, et al (2005) CD4+CD25+ regulatory lymphocytes induce regression of intestinal tumors in ApcMin/+ mice. Cancer Res 65: 3998–4004. 15899788 10.1158/0008-5472.CAN-04-3104

[40] Erdman SE, Rao VP, Olipitz W, Taylor CL, Jackson EA, et al (2010) Unifying roles for regulatory T cells and inflammation in cancer. Int J Cancer 126: 1651–1665. 19795459 10.1002/ijc.24923 PMC4068029

[41] Erdman SE, Rao VP, Poutahidis T, Ihrig MM, Ge Z, et al (2003) CD4(+)CD25(+) regulatory lymphocytes require interleukin 10 to interrupt colon carcinogenesis in mice. Cancer Res 63: 6042–6050. 14522933

[42] Zhang YL, Li J, Mo HY, Qiu F, Zheng LM, et al (2010) Different subsets of tumor infiltrating lymphocytes correlate with NPC progression in different ways. Mol Cancer 9: 4. 20064222 10.1186/1476-4598-9-4 PMC2818695

[43] Hooper SJ, Wilson MJ, Crean SJ (2009) Exploring the link between microorganisms and oral cancer: a systematic review of the literature. Head Neck 31: 1228–1239. 19475550 10.1002/hed.21140

[44] Elpek KG, Lacelle C, Singh NP, Yolcu ES, Shirwan H (2007) CD4+CD25+ T regulatory cells dominate multiple immune evasion mechanisms in early but not late phases of tumor development in a B cell lymphoma model. J Immunol 178: 6840–6848. 17513732 10.4049/jimmunol.178.11.6840

[45] Blatner NR, Bonertz A, Beckhove P, Cheon EC, Krantz SB, et al (2010) In colorectal cancer mast cells contribute to systemic regulatory T-cell dysfunction. Proceedings of the National Academy of Sciences of the United States of America 107: 6430–6435. 20308560 10.1073/pnas.0913683107 PMC2851977

[46] Kryczek I, Banerjee M, Cheng P, Vatan L, Szeliga W, et al (2009) Phenotype, distribution, generation, and functional and clinical relevance of Th17 cells in the human tumor environments. Blood 114: 1141–1149. 19470694 10.1182/blood-2009-03-208249 PMC2723011

[47] Kryczek I, Wei S, Szeliga W, Vatan L, Zou W (2009) Endogenous IL-17 contributes to reduced tumor growth and metastasis. Blood 114: 357–359. 19289853 10.1182/blood-2008-09-177360 PMC2714210

[48] Grossman WJ, Verbsky JW, Barchet W, Colonna M, Atkinson JP, et al (2004) Human T regulatory cells can use the perforin pathway to cause autologous target cell death. Immunity 21: 589–601. 15485635 10.1016/j.immuni.2004.09.002

[49] Koenen HJ, Smeets RL, Vink PM, van Rijssen E, Boots AM, et al (2008) Human CD25highFoxp3pos regulatory T cells differentiate into IL-17-producing cells. Blood 112: 2340–2352. 18617638 10.1182/blood-2008-01-133967

[50] Benchetrit F, Ciree A, Vives V, Warnier G, Gey A, et al (2002) Interleukin-17 inhibits tumor cell growth by means of a T-cell-dependent mechanism. Blood 99: 2114–2121. 11877287 10.1182/blood.v99.6.2114

[51] Banerjea A, Bustin SA, Dorudi S (2005) The immunogenicity of colorectal cancers with high-degree microsatellite instability. World J Surg Oncol 3: 26. 15890075 10.1186/1477-7819-3-26 PMC1166579

[52] Ribic CM, Sargent DJ, Moore MJ, Thibodeau SN, French AJ, et al (2003) Tumor microsatellite-instability status as a predictor of benefit from fluorouracil-based adjuvant chemotherapy for colon cancer. N Engl J Med 349: 247–257. 12867608 10.1056/NEJMoa022289 PMC3584639

[53] Fontenot JD, Rudensky AY (2005) A well adapted regulatory contrivance: regulatory T cell development and the forkhead family transcription factor Foxp3. Nat Immunol 6: 331–337. 15785758 10.1038/ni1179

[54] Walker MR, Kasprowicz DJ, Gersuk VH, Benard A, Van Landeghen M, et al (2003) Induction of FoxP3 and acquisition of T regulatory activity by stimulated human CD4+CD25- T cells. J Clin Invest 112: 1437–1443. 14597769 10.1172/JCI19441 PMC228469

[55] Baron U, Floess S, Wieczorek G, Baumann K, Grutzkau A, et al (2007) DNA demethylation in the human FOXP3 locus discriminates regulatory T cells from activated FOXP3(+) conventional T cells. Eur J Immunol 37: 2378–2389. 17694575 10.1002/eji.200737594

