# Supplementary figures and images for: Prognostic Impact of FoxP3+ Regulatory T Cells in Relation to CD8+ T Lymphocyte Density in Human Colon Carcinomas
Source: PLoS One. 2012 Aug 6;7(8):e42274. doi: 10.1371/journal.pone.0042274 (PMC3412852; doi:10.1371/journal.pone.0042274)

**Figure S1**

(a)

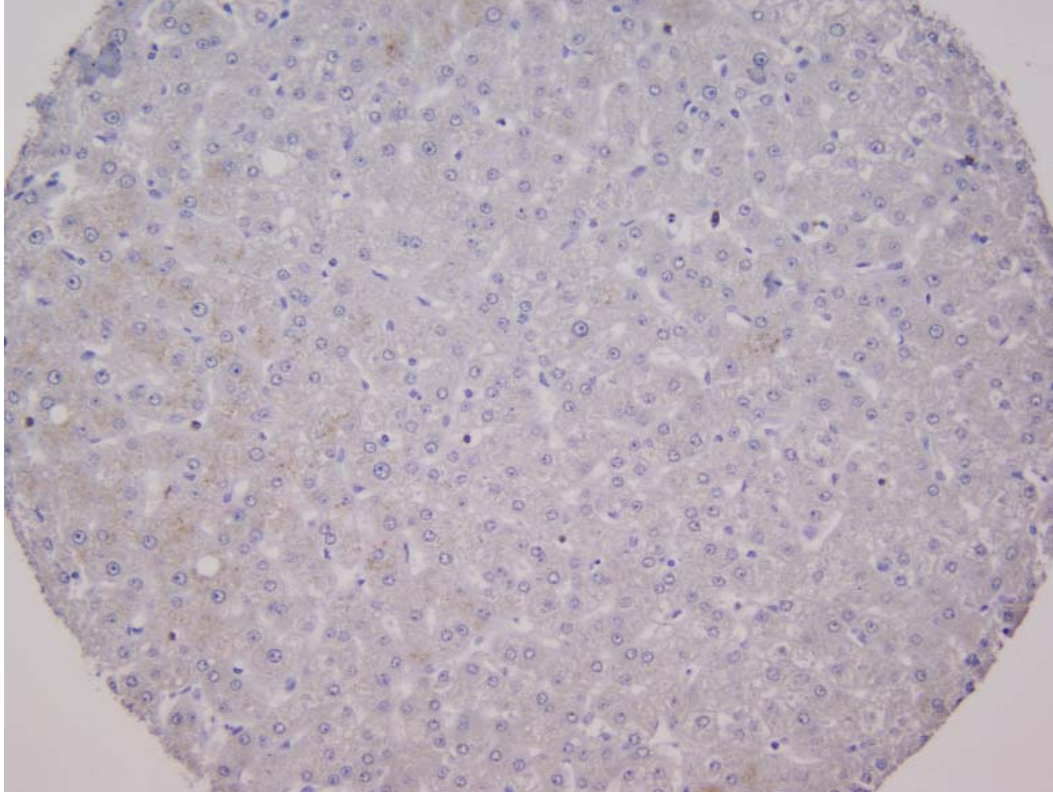

(b)

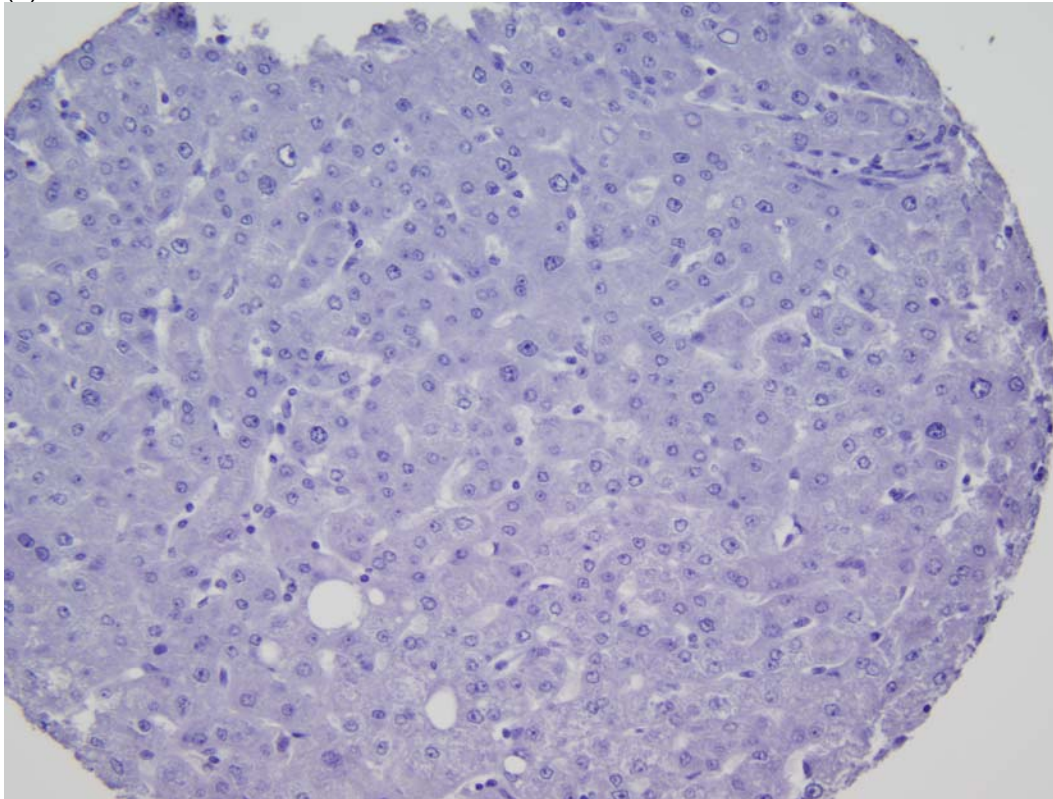

(c)

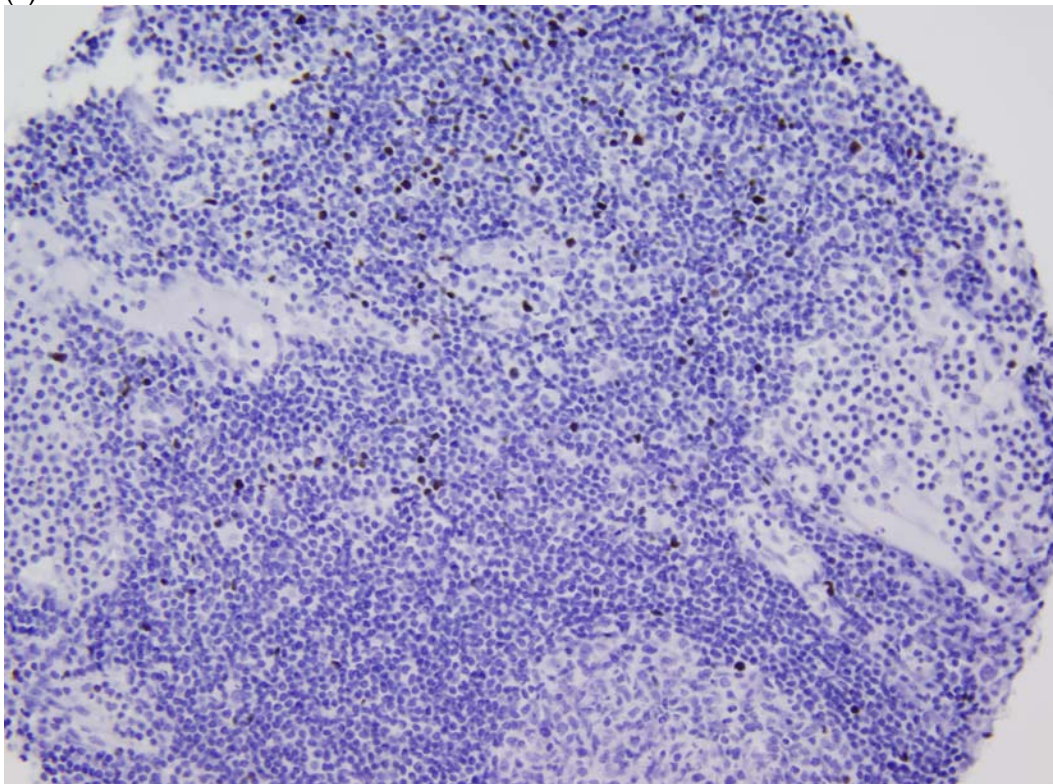

Supplement: Figure S1 — Control images for CD8+ and FoxP3+ staining. Normal human liver is shown as a negative control for CD8+ (a) and FoxP3+ (b) T cell immunostaining, and tonsil tissue (c) was stained as a positive control for FoxP3+ T cells (20× magnification). (PDF) [file pone.0042274.s001.pdf]
