# Supplementary material for: Prognostic Impact of FoxP3+ Regulatory T Cells in Relation to CD8+ T Lymphocyte Density in Human Colon Carcinomas
Source: PLoS One. 2012 Aug 6;7(8):e42274. doi: 10.1371/journal.pone.0042274 (PMC3412852; doi:10.1371/journal.pone.0042274)
Supplement: Table S1 — Comparison of Clinicopathologic Variables by Mismatch Repair Status. (DOC) [file pone.0042274.s002.doc]

**Table S1. Comparison of Clinicopathologic Variables by Mismatch Repair Status**

| **Clinicopathologic Variable** |  |  |  | **Mismatch Repair** | | | ***P*** |
| --- | --- | --- | --- | --- | --- | --- | --- |
|  |  | **TOTAL** |  | **Deficient** | **Proficient** | |  |
|  |  | **(N=183)^a^** |  | **(N=22)** | **(N=161)** | |  |
| **Gender**, N (%) |  |  |  |  | |  | 0.0751 |
| Female |  | 84 (46%) |  | 14 (64%) | | 70 (44%) |  |
| Male |  | 99 (54%) |  | 8 (36%) | | 91 (56%) |  |
| **Stage**, N (%) |  |  |  |  | |  | 0.9845 |
| II |  | 33 (18%) |  | 4 (18%) | | 29 (18%) |  |
| III |  | 150 (82%) |  | 18 (82%) | | 132 (82%) |  |
| **Histologic Grade**, N (%) |  |  |  |  | |  | 0.0005 |
| Well or moderate differentiation |  | 119 (65%) |  | 7 (32%) | | 112 (70%) |  |
| Poorly or undifferentiated |  | 64 (35%) |  | 15 (68%) | | 49 (30%) |  |
| **Tumor Site**, N (%) |  |  |  |  | |  | 0.0007 |
| Distal |  | 87 (48%) |  | 3 (14%) | | 84 (52%) |  |
| Proximal |  | 96 (52%) |  | 19 (86%) | | 77 (48%) |  |

^a^ Samples for which mismatch repair and immune marker data are available.
