# Supplementary material for: Prognostic Impact of FoxP3+ Regulatory T Cells in Relation to CD8+ T Lymphocyte Density in Human Colon Carcinomas
Source: PLoS One. 2012 Aug 6;7(8):e42274. doi: 10.1371/journal.pone.0042274 (PMC3412852; doi:10.1371/journal.pone.0042274)
Supplement: Table S2 — Density of T Lymphocyte Infiltration in Different Locations per Tumor Core (0.28 mm2 surface area) in Colon Cancer (N = 216). (DOC) [file pone.0042274.s003.doc]

| **Table S2. Density of T Lymphocyte Infiltration in Different Locations per Tumor Core (0.28 mm^2^ surface area) in Colon Cancer (N = 216)** | | | | | |
| --- | --- | --- | --- | --- | --- |
| **T cell type and location** |  | **Median** | **Mean** | **Interquartile range** | ***P*** |
| **CD8+ cytotoxic** |  |  |  |  |  |
| Epithelial |  | 11 | 24 | 2–3 | <.0001 |
| Stromal |  | 20 | 37 | 3–53 |  |
| **FoxP3+ regulatory** |  |  |  |  |  |
| Epithelial |  | 10 | 25 | 3–24 | 0.199 |
| Stromal |  | 12 | 21 | 0–31 |  |
